# The clinical and financial cost of mental disorders among elderly patients with gastrointestinal malignancies

**DOI:** 10.1002/cam4.3509

**Published:** 2020-10-06

**Authors:** Jeremy P. Harris, Mehr Kashyap, Jessica N. Humphreys, Erqi L. Pollom, Daniel T. Chang

**Affiliations:** ^1^ Department of Radiation Oncology University of California Irvine, Orange CA USA; ^2^ Department of Radiation Oncology Stanford University Stanford CA USA; ^3^ Division of Palliative Medicine Department of Medicine University of California San Francisco CA USA

**Keywords:** colon cancer, financial toxicity, gastrointestinal cancer, mental disorder, SEER‐Medicare

## Abstract

The clinical and financial effects of mental disorders are largely unknown among gastrointestinal (GI) cancer patients. Using the Surveillance, Epidemiology, and End Results (SEER)‐Medicare linked database, we identified patients whose first cancer was a primary colorectal, pancreatic, gastric, hepatic/biliary, esophageal, or anal cancer as well as those with coexisting depression, anxiety, psychotic, or bipolar disorder. Survival, chemotherapy use, total healthcare expenditures, and patient out‐of‐pocket expenditures were estimated and compared based on the presence of a mental disorder. We identified 112,283 patients, 23,726 (21%) of whom had a coexisting mental disorder. Median survival for patients without a mental disorder was 52 months (95% CI 50–53 months) and for patients with a mental disorder was 43 months (95% CI 42–44 months) (*p* < 0.001). Subgroup analysis identified patients with colorectal, gastric, or anal cancer to have a significant association between survival and presence of a mental disorder. Chemotherapy use was lower among patients with a mental disorder within regional colorectal cancer (43% vs. 41%, *p* = 0.01) or distant colorectal cancer subgroups (71% vs. 63%, *p* < 0.0001). The mean total healthcare expenditures were higher for patients with a mental disorder in first year following the cancer diagnosis (increase of $16,823, 95% CI $15,777‐$18,173), and mean patient out‐of‐pocket expenses were also higher (increase of $1,926, 95% CI $1753–$2091). There are a substantial number of GI cancer patients who have a coexisting mental disorder, which is associated with inferior survival, higher healthcare expenditures, and greater personal financial burden.

## Graphical abstract

In this SEER‐Medicare study of GI cancers, the 21% of patients with a pre‐existing mental disorder had inferior overall survival outcomes (52 months vs 43 months, *p* < 0.001). Patients with mental disorders also had increased healthcare expenditures of $16,000 in the first year of diagnosis and higher personal financial burden.

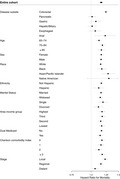

## 1 INTRODUCTION

Gastrointestinal (GI) malignancies encompass a wide array of cancer subsites, histologies, and prognoses. Uniting these cancers is an increased burden of symptoms related to the diseases and treatments, which in turn results increased rates of hospitalization.^1^ In response, oncologists are becoming increasingly aware of the importance of supportive care for managing patients. ASCO recognizes that supportive care measures are paramount to delivering comprehensive oncologic care, and current recommendations advocate for early palliative care interventions for many patients.^2^ Due to the high rate of psychiatric symptoms for these patients, psycho‐oncologic assessments are an important piece of the supportive care measures.

With regards to GI malignancies, it has historically been suggested that mental disorders are less prevalent.^3^ However, more recent studies have disputed that claim, finding rates of 18‐29% of patients with a pre‐existing mental disorder.^4^, ^5^, ^6^, ^7^ Thus, developing a better understanding of the impact of having a mental disorder comorbid with a GI cancer is needed. We set out to determine the clinical and financial implications of having a comorbid mental disorder for elderly patients with GI malignancies in the United States.

## 2 METHODS

### 2.1 Data source

We employed the Surveillance, Epidemiology, and End Results (SEER)‐Medicare linked database and identified patients whose first cancer was a primary colorectal, pancreatic, gastric, hepatic/biliary, esophageal, or anal cancer. SEER‐Medicare is a large population‐based linked‐dataset maintained by the National Cancer Institute. It details inpatient and outpatient medical claims from Medicare beneficiaries who have cancer and are included in the SEER database, which is a registry that covers approximately 34% of the U.S. population.^8^, ^9^

### 2.2 Patient selection

The cohort included patients diagnosed 2004‐2013 who were at least 65 years old with Medicare Part A and B enrollment without HMO coverage starting 12 months prior to cancer diagnosis to ensure completeness of claims data. We excluded patients with missing or incomplete age, stage, date of diagnosis, or follow up/death information. Censoring occurred at the end of December 2014 or when Medicare coverage was lost.

### 2.3 Mental disorder identification

Medicare billing claims were used to identify patients with mental disorders. We included patients with depression disorders (ICD9 diagnosis codes 296.2‐296.36, 300.4, 311), anxiety disorders (ICD9 diagnosis codes 293.84, 300‐300.09, 300.10, 300.2‐300.3, 300.5, 308‐308.9, 309.81, 313.0), psychotic disorders (ICD9 diagnosis codes 293.81‐293.82, 295‐295.95, 297‐298.9), and bipolar disorders (ICD9 diagnosis codes 296.0‐296.16, 296.4‐296.99). A coexisting mental disorder was defined as a condition identified in any billing claim 12 months prior to 6 months following the GI cancer diagnosis. To reduce immortal time bias, only patients living at least 6 months after cancer diagnosis were included in this study.

### 2.4 Demographic and clinical characteristics

Using data from the SEER registry, we categorized the following demographics: age, sex, race, Hispanic ethnicity, marital status, and area income. We determined whether patients had dual Medicaid, which can identify patients who are considered low‐income. Clinical characteristics identified from SEER data were cancer stage (grouped by local, regional, or distant according to SEER summary staging), and disease subsite.^10^ A Charlson comorbidity index modified for cancer patients was used as a proxy for overall comorbidity burden and was determined through inpatient and outpatient medical claims.^11^, ^12^

### 2.5 Endpoint identification

Overall survival (OS) was the time from diagnosis to death, with censoring occurring at the end of December 2014. Cause of death data were used to determine cancer‐specific mortality (CSM). Total inpatient and outpatient healthcare expenditures were estimated from a summation of a patient's Medicare Part A and Part B reimbursements from Medicare and third‐party payers and patient payments. Cost estimates included those from all hospital and outpatient associated claims. Patient out‐of‐pocket expenses were the total deductible and coinsurance costs for Medicare Part A and Part B claims. Prices were converted to 2019 dollars by using the Consumer Price Index.^13^ Use of chemotherapy or radiation therapy was identified within 6 months of cancer diagnosis through Medicare claims and included intravenously and orally administered drugs.^14^ Site‐specific surgical intervention was classified by local excision/ablation or surgical resection according to SEER classifications.

### 2.6 Statistical Analysis

Clinical and demographic characteristics were compared between patients with or without a coexisting mental disorder using chi‐squared tests. Survival was estimated with Kaplan‐Meier methods, and between group comparisons were made with log‐rank testing. Between group survival comparisons were also made using propensity score matched cohorts to control for confounding variables. Cumulative incidence estimates were used to determine CSM rates with non‐cancer deaths defined as competing events, and between group comparisons were made with Gray's tests. Propensity scores were determined from a logistic regression for the odds of having a mental disorder using age, sex, race, ethnicity, marital status, area income, dual Medicaid status, cancer stage, Charlson comorbidity index, and disease subsite. 1:1 matching was done with a nearest‐neighbor technique with a maximum caliper of 0.05 times the standard deviation of the logit of the propensity score.^15^, ^16^ Balance was assessed with a 10% maximum standardized difference.^17^

Individual subgroup analysis of mortality was performed with multivariable proportional hazards models. Adjustments were made for age, sex, race, ethnicity, marital status, area income, dual Medicaid status, cancer stage, Charlson comorbidity index, and disease subsite. The proportional hazards assumption was evaluated using log‐log plots and Schoenfeld residuals.

Propensity score matching was also done for individual subgroups by cancer subsite and stage. Within these subgroups, OS and the use of chemotherapy was compared based on the presence of a mental disorder using log‐rank and McNemar's tests, respectively.

The unadjusted per‐year total healthcare spending was compared between groups with Mann‐Whitney U testing. Patients needed Medicare claims data for each year to be included in that year's cost assessment. Adjusted annual estimates for total and patient out‐of‐pocket expenditures were made using a two‐part model because the cost data was right skewed and there were a portion of zero values.^18^ The first part was a multivariable logistic regression for having any non‐zero costs. The second part was a generalized linear model for the mean cost conditional on there being greater than zero cost and using a gamma distribution with a log link.^19^ Differences in mean total and patient out‐of‐pocket costs were determined based on mental disorder status. Bootstrapping methods were used to estimate confidence intervals in differences.

Statistical significance was set at 0.05, and all tests were two‐tailed. Data analysis and statistical testing was performed using R (version 3.5, R Foundation for Statistical Computing, Vienna, Austria).

## 3 RESULTS

We identified 112,283 patients who met inclusion criteria (Figure S1). Represented cancer subsites were colorectal (*n* = 60283), pancreatic (*n* = 7977), gastric (*n* = 7204), hepatic/biliary (*n* = 7513), esophageal (*n* = 4158), and anal cancer (*n* = 1422). There were 23,726 patients (21%) with a coexisting mental disorder, composed of 16,138 (21%) of the colorectal cancer cohort, 2,401 (23%) of the pancreatic cancer cohort, 1,792 (20%) of the gastric cancer cohort, 1,891 (20%) of the hepatic/biliary cancer cohort, 1,001 (19%) of the esophageal cancer cohort, and 503 (26%) of the anal cancer cohort (Table 1). There were 15,091 patients (13%) with coexisting depression, 11,180 patients (10%) with coexisting anxiety, 3594 patients (3%) with coexisting psychotic, and 1052 patients (0.9%) with coexisting bipolar disorders (Tables S1‐S4).

**Table 1:** Demographic and clinical characteristics according to coexisting mental disorder status.

	No mental disorder		Diagnosed mental disorder		*p* value
	n	%	n	%	
Disease subsite					
Colorectal	60283	68%	16138	68%	<0.0001
Pancreatic	7977	9%	2401	10%	
Gastric	7204	8%	1792	8%	
Hepatic/biliary	7513	8%	1891	8%	
Esophageal	4158	5%	1001	4%	
Anal	1422	2%	503	2%	
Age group					
65–74	39687	45%	10105	43%	<0.0001
75–84	36474	41%	9686	41%	
≥85	12396	14%	3935	17%	
Sex					
Male	46484	52%	8767	37%	<0.0001
Female	42073	48%	14959	63%	
Race					
White	72890	82%	20873	88%	<0.0001
Black	8096	9%	1778	7%	
Asian/Pacific Islander	6882	8%	926	4%	
North American Native	374	0%	82	0%	
Unknown	315	0%	67	0%	
Ethnicity					
Non‐Hispanic	82625	93%	22140	93%	0.95
Hispanic	5932	7%	1586	7%	
Marital status					
Married/domestic partner	48436	55%	10264	43%	<0.0001
Widowed	22854	26%	7731	33%	
Single	7184	8%	2280	10%	
Separated/divorced	6427	7%	2357	10%	
Unknown	3656	4%	1094	5%	
Area income					
Highest	21408	24%	5265	22%	<0.0001
Third	20451	23%	5408	23%	
Second	19730	22%	5612	24%	
Lowest	18752	21%	5755	24%	
Unknown	8216	9%	1686	7%	
Dual Medicaid status					
No	70008	79%	16274	69%	<0.0001
Yes	18549	21%	7452	31%	
Charlson comorbidity index					
0	41169	46%	8229	35%	<0.0001
1	22492	25%	6037	25%	
2	11973	14%	3891	16%	
≥3	12923	15%	5569	23%	
Stage					
Local	38832	44%	10326	44%	<0.0001
Regional	35476	40%	9970	42%	
Distant	14249	16%	3430	14%	

**Figure 1:**
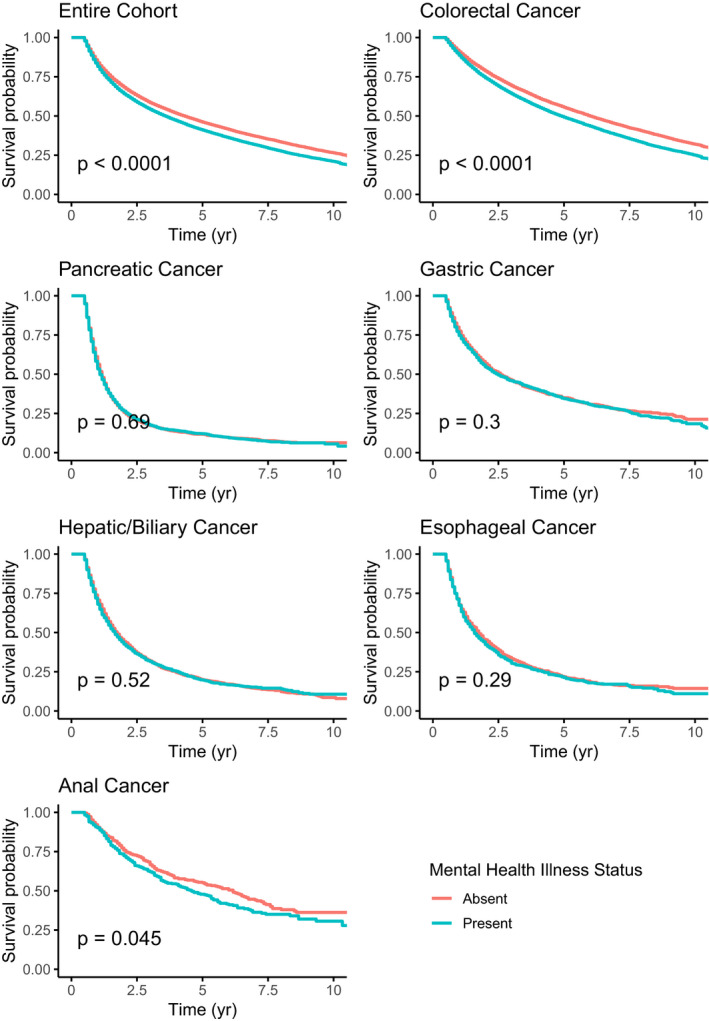
Overall survival according to mental disorder status. Cohorts were obtained through propensity score matching

After propensity score‐based matching, median survival for patients without a mental disorder was 52 months (95% CI 50–53 months) and for patients with a mental disorder was 43 months (95% CI 42‐44 months, *p* < 0.001, Figure 1). Subgroup analysis by disease site was done with propensity score‐based matching and demonstrated a statistically significant difference in survival according to mental disorder status for patients with colorectal (median OS 72 months [95% CI 70–74 months] vs 59 months [95% CI 57–61 months], *p* < 0.0001) and anal cancer (median OS 74 months [95% CI 61–85 months] vs 55 months [95% CI 46–65 months], *p* = 0.045). Results were similar with unmatched, raw data (Figure S2).

**Figure 2:**
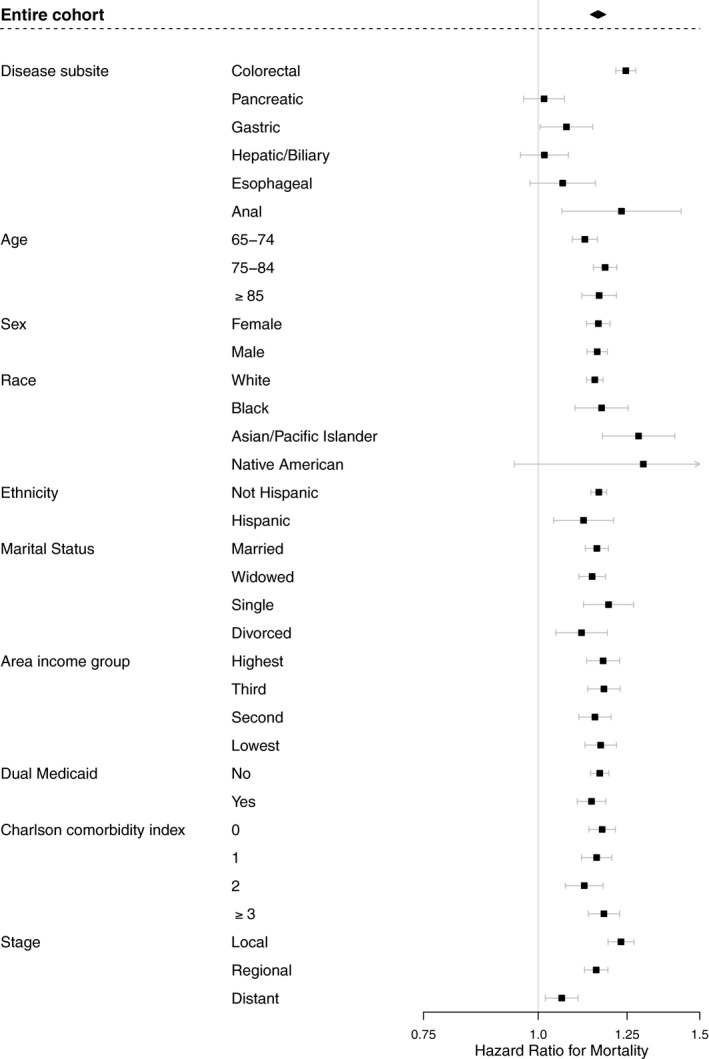
Association between mental disorder status and overall survival by individual subgroup. Each cohort represents findings from individual subgroup multivariable analysis

On multivariable analysis, having a mental disorder was associated with inferior overall survival (HR 1.16, 95% CI 1.14–1.18). Individual subgroup analysis found a similar relationship for patients with colorectal (HR 1.25, 95% CI 1.22–1.28), gastric (HR 1.07, 95% CI 1.00–1.15), and anal cancer (HR 1.23, 95% CI 1.06‐1.43) (Figure 2). According to disease stage, the biggest association between mental disorder and survival was for local disease (HR 1.23, 95% CI 1.19–1.27), followed by regional disease (HR 1.16, 95% CI 1.12–1.19), followed by distant disease (HR 1.06, 95% CI 1.02–1.11).

Propensity score‐based matching was done on individual stage subgroups for patients with colorectal, gastric, and anal cancer, for whom survival differed based on mental disorder status. A significant decrease in OS was associated with having a mental disorder for subgroups of patients with local or regional colorectal cancer or regional gastric cancer only (Figure S3).

After propensity score‐based matching, 5‐year CSM was 30% for patients without a mental disorder and 31% for those with a mental disorder (*p* = 0.03). Subgroup analysis by disease site was done with propensity score‐based matching and demonstrated a statistically significant increase in CSM for patients with a mental disorder and colorectal cancer (5‐year incidence of 20% vs 21%, *p* < 0.0001), but a decrease in CSM for patients with pancreatic cancer (5‐year incidence of 80% vs 77%, *p* = 0.03) (Figure S4). Directional patterns were similar with unmatched, raw data, but there was also a decrease in CSM for patients with a mental disorder and gastric cancer (5‐year incidence of 37% vs 32%, *p* = 0.003) (Figure S5).

Chemotherapy use differed for patients with regional colorectal cancer (43% vs 41% with a mental disorder, *p* = 0.01), distant colorectal cancer (71% vs 63% with a mental disorder, *p* < 0.0001), regional pancreatic cancer (71% vs 67% with a mental disorder, *p* = 0.03), regional esophageal cancer (73% vs 80% with a mental disorder, *p* = 0.03), local anal cancer (56% vs 69% with a mental disorder, *p* = 0.004), and regional anal cancer (67% vs 80% with a mental disorder, *p* = 0.04) (Table S5). Radiation therapy was more likely to be employed for patients with a mental disorder and any stage colorectal, distant pancreatic, local gastric, distant gastric, local hepatic/biliary, regional hepatic/biliary, and local anal cancer (Table S6). Patients with mental disorders were also less likely to undergo local excision/ablation for local colorectal cancer (14% vs 12%, *p* = 0.0006) (Table S7). Finally, having a mental disorder made it less likely for patients to undergo local excision/ablation for local gastric cancer (16% vs 15%), but more likely to have a larger surgical resection (53% vs 60%, *p* = 0.02).

**Figure 3:**
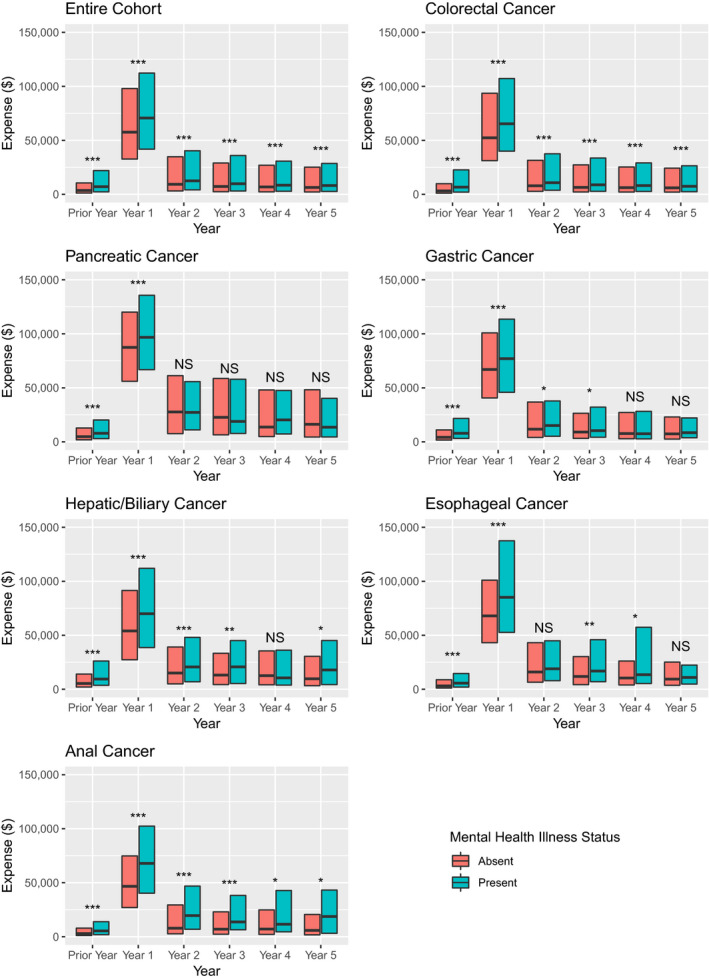
Median total unadjusted healthcare expenditures according to year of cancer diagnosis and mental disorder status. Solid lines represent median values, with box hinges showing the interquartile ranges. Statistical significance is indicated by “*” at the 0.05 level, “**” at the 0.01 level, and “***” at the 0.001 level, and “NS” indicates nonsignificant differences.

The median total inpatient and outpatient unadjusted healthcare expenditures were higher for patients without and with a mental disorder in each year studied (Figure 3). Two‐part models for the mean total healthcare expenditures determined that costs were higher for patients with a mental disorder in the year prior to (increase of $8873, 95% CI $8295‐$9414), first year following (increase of $16,823, 95% CI $15,777‐$18,173), and second year following the cancer diagnosis (increase of $837, 95% CI $434‐$1206) (Table 2). There were not significant differences in the third to fifth year following the cancer diagnosis. Similarly, mean patient out‐of‐pocket expenses were higher for patients with a mental disorder in the year prior to (increase of $1085, 95% CI $1035‐$1132), first year following (increase of $1926, 95% CI $1753‐$2091), and second year following the cancer diagnosis (increase of $109, 95% CI $62–$161).

**Table 2:** Difference in mean expenditures for inpatient and outpatient encounters according to mental disorder status. Positive numbers indicate higher expenses associated with having a mental disorder

	Total healthcare expenditures				Patient out‐of‐pocket expenditures			
	Mental disorder absent	Mental disorder present	Change (increase or decrease) with a mental disorder		Mental disorder absent	Mental disorder present	Change (increase or decrease) with a mental disorder	
	($)	($)	($)	95% Confidence Interval ($)	($)		($)	95% Confidence Interval ($)
Year prior to diagnosis	$10,307	$19,181	$8873	($8295 to $9414)	$1517	$2,602	$1,085	($1035 to $1132)
Year 1	$70,157	$86,980	$16,823	($15,777 to $18,173)	$9009	$10,936	$1,926	($1753 to $2091)
Year 2	$10,669	$11,507	$837	($434 to $1,206)	$1679	$1,788	$109	($62 to $161)
Year 3	$4,811	$4,937	$127	(‐$91 to $355)	$759	$757	‐$2	(‐$36 to $31)
Year 4	$2,495	$2,328	‐$167	(‐$310 to ‐$34)	$390	$358	‐$31	(‐$51 to ‐$9)
Year 5	$1,316	$1,097	‐$219	(‐$289 to ‐$121)	$202	$166	‐$36	(‐$45 to ‐$24)

## 4 DISCUSSION

In this study of an elderly cohort, 21% of patients were identified with a coexisting mental disorder, which was associated with a decrease in median OS from 52 months to 43 months and slight increase in 5‐year CSM from 30% to 31%. This association was most dramatic for patients with local disease at presentation, as well as the subgroups with colorectal or anal cancer. In addition, having a mental disorder was associated with a decreased chance of receiving chemotherapy for regional or distant stage colorectal cancer, decreased probability of having a local excision/ablation for local colorectal cancer, and increased probability of receiving radiation therapy for many groups. Finally, total inpatient and outpatient healthcare expenditures were an average of $16,000 higher for patients with mental disorders in the first year following a cancer diagnosis, which consisted of an increase of $2,000 in patient out‐of‐pocket costs.

Our finding of the substantial health outcome disparity with having a mental disorder is consistent with prior studies of patients with GI malignancies.^20^, ^21^ Some proposed explanations have included that in general, patients with psychiatric illnesses can be more likely to have advanced cancer at the time of presentation, less likely to undergo surgery, and more likely to have fewer cycles of chemotherapy.^20^ In the United States, studies have demonstrated that patients with colon cancer are less likely to receive definitive treatment, and those with rectal cancer are less likely to undergo a sphincter sparing surgery when they have a comorbid mental disorder.^4^, ^6^ Similarly, in the current study, we found that certain subgroups with colorectal or pancreatic cancer were less likely to undergo chemotherapy when they had a coexisting mental disorder, and those with local colorectal cancer were less likely to undergo local excision/ablation. Interestingly, patients with a mental disorder were in general more likely to receive radiation therapy. However, the intent of radiation therapy was not known in this study, which could make interpretation more challenging. For example, while the increased use of radiation therapy for patients with distant stage colorectal cancer may be consistent with attentiveness to symptomatic control, the increased use of radiation therapy for those with local or regional colorectal cancer could imply the lack of timely definitive treatment, more aggressive tumors with higher probability of progression, or even residual bias with more advanced cancers among patients with mental disorders. We would also point out that the large differences in OS were not fully explained by CSM, implying that comorbid health conditions or even mental health disorders were simultaneously driving the inferior outcomes for these patients.

There are many potential hypotheses for the decrease in chemotherapy or local excision/ablation that the current study and others have demonstrated. For example, a mental disorder could negatively affect treatment decisions made by the patient due to the underlying comorbidity or the psychopharmacologic agent.^22^ Additionally, there may be an unmet, increased need for better social support networks, which could exacerbate a decrease in recommended cancer treatment adherence related to the fact that treatment paradigms are challenging to navigate.^23^ Again, these explanations are consistent with our findings of inferior OS and CSM among some subgroups in the presence of a comorbid mental disorder. Lastly, there could be a potential biological explanation that mental disorders increase the body's stress response, which in turn could result in worse clinical outcomes.^24^, ^25^

Mental disorder management should be considered critical not only because of the therapeutic and oncologic outcome discrepancies identified in this study, but also because non‐cancer mortality rates were higher for these patients. In response, there has been interest in determining how to better manage the psychiatric symptoms for cancer patients. Psychotherapy and counseling have shown to improve depression symptoms and quality of life for cancer patients.^26^, ^27^, ^28^ It is also plausible that the resulting improved psychiatric symptom burden could decrease the body's stress response and result in improved survival.^29^ Medications have similarly shown a benefit, with one retrospective study showing an improvement in lung cancer‐specific survival and suggesting a biological role for certain tricyclic antidepressants or norepinephrine and dopamine reuptake inhibtors.^30^ Furthermore, this is an area where patients sometimes turn to complementary and alternative medicine, though the quality of supporting data is generally low.^31,32^

Additionally, for patients with mental disorders there have been investigations in improving cancer detection to reduce the stage distribution disparity. In the current study, patients with a mental disorder were slightly more likely to be diagnosed with regional disease. In one recent meta‐analysis, identified themes included implementing screening templates, staff education/training, automated physician prompting, and integration of psychiatric with physical healthcare.^33^ While these methods generally show some amount of success, there remain challenges from a resource, patient‐engagement, practitioner education/training, and workload standpoint. The Centers for Disease Control and Prevention (CDC) has recognized this problem and created the free, online Provider Education for Mental Health Care of Cancer Survivors Training, and through the National Comprehensive Cancer Control Program the CDC is funding projects addressing psychiatric symptoms among cancer survivors.^34^

With regards to the higher healthcare expenditures for patients with mental disorders that were identified in this study, these patients are more likely to have higher rates of healthcare utilization, and consequently, experience significant personal financial hardship due to incurred costs. Cancer patients with depression, for example, have more emergency department visits, overnight hospital admissions, 30‐day readmissions, and non‐mental health provider visits.^35^, ^36^ This increase in utilization translates into higher total healthcare costs, including inpatient, outpatient, prescription drug, and long‐term care costs.^37^ Of note, our findings showed that these increased costs were despite a lower utilization of chemotherapy or surgical intervention for some patients with colorectal and pancreatic cancer. However, most subgroups in the current study were more likely to receive radiation therapy, which could also be related to the increased costs. Higher costs of care are particularly unfavorable since cancer patients, in general, experience more financial hardship, such as difficulty paying medical bills and forgoing care due to cost compared to those without cancer.^38^

Providing mental health services could mitigate the financial burden associated with having a mental disorder, though evidence is currently limited and mixed. In one recent study, cancer patients with depression often had significantly lower annual healthcare costs when they were seen by mental health providers.^39^ Similarly, a Canadian prospective study of patients with early stage breast cancer found that patients who participated in cognitive behavioral psychosocial meetings had lower healthcare expenditures.^40^ Nevertheless, there is also contrary evidence that indicates treatments such as psychotherapy and antidepressants may not significantly impact long‐term healthcare expenditures in cancer patients with depression.^41^ Together, these studies demonstrate a need to better understand which patients will benefit most from psychiatric interventions and what services are most effective.

Limitations of the current study include that it is retrospective and naturally incurs selection bias. In addition, despite propensity‐score matching and multivariable economic modeling, there may be important unmeasured confounders that could bias the study. We utilized billing claims from the Medicare insurance program, which may underrepresent the true rate of mental disorders among cancer patients. Cause of death data in the registry may not be accurate, which can bias CSM analyses. Furthermore, these billing claims do not contain data on the cost or clinical effect of prescription or over‐the‐counter drug use, and additional studies of psychiatric medications especially are necessary to determine mental health treatment effects. In addition, given the wide range of cancer therapy including specific surgical procedures and type of radiation therapy and other ancillary care associated with these treatments, we were unable to further characterize sources of increased costs for patients with disorders. Finally, our cohort represented an elderly group of mostly white patients with GI cancers and may not be generalizable to younger or more diverse groups of patients.

## 5 CONCLUSIONS

In summary, GI cancer patients with comorbid mental disorders are more likely to have compromised overall and cancer‐specific survival outcomes, higher healthcare expenditures, and greater personal financial burden. Patients with regional or distant colorectal cancer were less likely to receive chemotherapy, those with local colorectal cancer were less likely to undergo local excision/ablation, and many groups were more likely to receive radiation therapy. Understanding the mental health of one's patient is crucial not only because of the significant rate and detrimental effects of having a mental disorder, but also because a number of therapeutic options are available. This study further highlights the importance of efforts such as the free mental health screening training for physicians made available by the CDC. Lastly, reducing personal costs remains an understudied, unmet need in health services research.

## Conflicts of Interest

No authors have any conflicts of interest to report for this work.

## Supporting information

Figures S1‐S5, Tables S1‐S7

## Data Availability

The data that support the findings of this study are available from the National Cancer Institute. Restrictions apply to the availability of these data, which were used under license for this study. Data are available at https://healthcaredelivery.cancer.gov/seermedicare/ with the permission of the National Cancer Institute.
