# Supplementary material for: The clinical and financial cost of mental disorders among elderly patients with gastrointestinal malignancies
Source: Cancer Med. 2020 Oct 6;9(23):8912–22. doi: 10.1002/cam4.3509 (PMC7724481; doi:10.1002/cam4.3509)
Supplement: Supplementary file 1 — Figures S1‐S5, Tables S1‐S7 [file CAM4-9-8912-s001.docx]

Supplemental

Figure S1. Cohort selection.

Table S1. Demographic and clinical characteristics according to coexisting depression disorder status.

| Disease subsite | No depression disorder | | Diagnosed depression disorder | | P value |
| --- | --- | --- | --- | --- | --- |
|  | n | % | n | % |  |
| Colorectal | 66275 | 68% | 10146 | 67% | <0.0001 |
| Pancreatic | 8797 | 9% | 1581 | 10% |  |
| Gastric | 7839 | 8% | 1157 | 8% |  |
| Hepatic/biliary | 8162 | 8% | 1242 | 8% |  |
| Esophageal | 4524 | 5% | 635 | 4% |  |
| Anal | 1595 | 2% | 330 | 2% |  |

Table S2. Demographic and clinical characteristics according to coexisting anxiety disorder status.

| Disease subsite | No anxiety disorder | | Diagnosed anxiety disorder | | P value |
| --- | --- | --- | --- | --- | --- |
|  | n | % | n | % |  |
| Colorectal | 68799 | 68% | 7622 | 68% | <0.0001 |
| Pancreatic | 9211 | 9% | 1167 | 10% |  |
| Gastric | 8152 | 8% | 844 | 8% |  |
| Hepatic/biliary | 8591 | 8% | 813 | 7% |  |
| Esophageal | 4666 | 5% | 493 | 4% |  |
| Anal | 1684 | 2% | 241 | 2% |  |

Table S3. Demographic and clinical characteristics according to coexisting psychotic disorder status.

| Disease subsite | No psychotic disorder | | Diagnosed psychotic disorder | | P value |
| --- | --- | --- | --- | --- | --- |
|  | n | % | n | % |  |
| Colorectal | 73795 | 68% | 2626 | 73% | <0.0001 |
| Pancreatic | 10134 | 9% | 244 | 7% |  |
| Gastric | 8769 | 8% | 227 | 6% |  |
| Hepatic/biliary | 9098 | 8% | 306 | 9% |  |
| Esophageal | 5036 | 5% | 123 | 3% |  |
| Anal | 1857 | 2% | 68 | 2% |  |

Table S4. Demographic and clinical characteristics according to coexisting bipolar disorder status.

| Disease subsite | No bipolar disorder | | Diagnosed bipolar disorder | | P value |
| --- | --- | --- | --- | --- | --- |
|  | n | % | n | % |  |
| Colorectal | 75694 | 68% | 727 | 69% | 0.15 |
| Pancreatic | 10281 | 9% | 97 | 9% |  |
| Gastric | 8921 | 8% | 75 | 7% |  |
| Hepatic/biliary | 9327 | 8% | 77 | 7% |  |
| Esophageal | 5111 | 5% | 48 | 5% |  |
| Anal | 1897 | 2% | 28 | 3% |  |

Figure S2. Overall survival according to mental disorder status. Data represent the entire cohort without matching.

Figure S3. Overall survival according to mental disorder status based on cancer stage subgroups for patients with colorectal, gastric, or anal cancer. Each subgroup represents cohorts from propensity score-based matching.

Figure S4. Cancer-specific mortality according to mental disorder status. Cohorts were obtained through propensity score matching.

Figure S5. Cancer-specific mortality according to mental disorder status. Data represent the entire cohort without matching.

Table S5. Use of any chemotherapy agent within 6 months from diagnosis of cancer according to the presence of a mental disorder. Each row represents data from a propensity score matched cohort.

|  |  |  | **No Mental Disorder Cohort** | | **Coexisting Mental Disorder Cohort** | |  |
| --- | --- | --- | --- | --- | --- | --- | --- |
|  |  |  | **Received Chemotherapy** | | **Received Chemotherapy** | |  |
| **Cancer Subtype** | **Stage** | **Size of 1:1 matched cohort** | **n** | **(%)** | **n** | **(%)** | **P value** |
| Colorectal | Local | 7423 | 721 | (10%) | 727 | (10%) | 0.89 |
|  | Regional | 6770 | 2899 | (43%) | 2755 | (41%) | 0.01 |
|  | Distant | 1836 | 1295 | (71%) | 1148 | (63%) | <0.0001 |
| Pancreatic | Local | 410 | 161 | (39%) | 150 | (37%) | 0.47 |
|  | Regional | 1206 | 853 | (71%) | 803 | (67%) | 0.03 |
|  | Distant | 726 | 548 | (75%) | 547 | (75%) | 1.00 |
| Gastric | Local | 858 | 131 | (15%) | 136 | (16%) | 0.80 |
|  | Regional | 624 | 343 | (55%) | 349 | (56%) | 0.77 |
|  | Distant | 284 | 198 | (70%) | 183 | (64%) | 0.22 |
| Hepatic/Biliary | Local | 894 | 284 | (32%) | 253 | (28%) | 0.12 |
|  | Regional | 718 | 265 | (37%) | 277 | (39%) | 0.55 |
|  | Distant | 249 | 142 | (57%) | 136 | (55%) | 0.63 |
| Esophageal | Local | 345 | 148 | (43%) | 145 | (42%) | 0.88 |
|  | Regional | 393 | 287 | (73%) | 313 | (80%) | 0.03 |
|  | Distant | 228 | 189 | (83%) | 184 | (81%) | 0.63 |
| Anal | Local | 277 | 156 | (56%) | 190 | (69%) | 0.004 |
|  | Regional | 138 | 93 | (67%) | 110 | (80%) | 0.04 |
|  | Distant | 46 | 35 | (76%) | 37 | (80%) | 0.81 |

Table S6. Use of radiation therapy within 6 months from diagnosis of cancer according to the presence of a mental disorder. Each row represents data from a propensity score matched cohort.

|  |  |  | **No Mental Disorder Cohort** | | **Coexisting Mental Disorder Cohort** | |  |
| --- | --- | --- | --- | --- | --- | --- | --- |
|  |  |  | **Received Radiotherapy** | | **Received Radiotherapy** | |  |
| **Cancer Subtype** | **Stage** | **Size of 1:1 matched cohort** | **n** | **(%)** | **n** | **(%)** | **P value** |
| Colorectal | Local | 7423 | 2657 | (36%) | 3309 | (45%) | <0.0001 |
|  | Regional | 6770 | 3338 | (49%) | 3765 | (56%) | <0.0001 |
|  | Distant | 1836 | 856 | (47%) | 952 | (52%) | 0.001 |
| Pancreatic | Local | 410 | 184 | (45%) | 204 | (50%) | 0.19 |
|  | Regional | 1206 | 768 | (64%) | 798 | (66%) | 0.21 |
|  | Distant | 726 | 231 | (32%) | 317 | (44%) | <0.0001 |
| Gastric | Local | 858 | 365 | (43%) | 432 | (50%) | 0.001 |
|  | Regional | 624 | 461 | (74%) | 474 | (76%) | 0.44 |
|  | Distant | 284 | 138 | (49%) | 170 | (60%) | 0.01 |
| Hepatic/Biliary | Local | 894 | 248 | (28%) | 298 | (33%) | 0.01 |
|  | Regional | 718 | 293 | (41%) | 364 | (51%) | 0.0003 |
|  | Distant | 249 | 108 | (43%) | 116 | (47%) | 0.53 |
| Esophageal | Local | 345 | 205 | (59%) | 231 | (67%) | 0.06 |
|  | Regional | 393 | 336 | (85%) | 351 | (89%) | 0.12 |
|  | Distant | 228 | 157 | (69%) | 153 | (67%) | 0.77 |
| Anal | Local | 277 | 201 | (73%) | 239 | (86%) | <0.0001 |
|  | Regional | 138 | 117 | (85%) | 127 | (92%) | 0.10 |
|  | Distant | 46 | 37 | (80%) | 41 | (89%) | 0.42 |

Table S7. Surgical resection or local excision according to the presence of a mental disorder. Each row represents data from a propensity score matched cohort. Cells with fewer than 10 patients are hidden to protect identity.

|  |  |  | **No Mental Disorder Cohort** | | | | **Coexisting Mental Disorder Cohort** | | | |  |
| --- | --- | --- | --- | --- | --- | --- | --- | --- | --- | --- | --- |
|  |  |  | **Local Excision/Ablation** | | **Resection** | | **Local Excision/Ablation** | | **Resection** | |  |
| **Cancer Subtype** | **Stage** | **Size of 1:1 matched cohort** | **n** | **(%)** | **n** | **(%)** | **n** | **(%)** | **n** | **(%)** | **P value** |
| Colorectal | Local | 7423 | 1032 | (14%) | 5933 | (80%) | 862 | (12%) | 6049 | (81%) | 0.0006 |
|  | Regional | 6770 | 34 | (1%) | 6544 | (97%) | 36 | (1%) | 6529 | (96%) | 0.87 |
|  | Distant | 1836 | 25 | (1%) | 1258 | (69%) | 19 | (1%) | 1304 | (71%) | 0.14 |
| Pancreatic | Local | 410 | <10 |  | 136 | (33%) | <10 |  | 135 | (33%) | 0.16 |
|  | Regional | 1206 | <10 |  | 604 | (50%) | <10 |  | 655 | (54%) | 0.18 |
|  | Distant | 726 | <10 |  | 63 | (9%) | <10 |  | 87 | (12%) | 0.14 |
| Gastric | Local | 858 | 134 | (16%) | 452 | (53%) | 127 | (15%) | 515 | (60%) | 0.02 |
|  | Regional | 624 | <10 |  | 505 | (81%) | <10 |  | 501 | (80%) | 0.72 |
|  | Distant | 284 | <10 |  | 67 | (24%) | <10 |  | 63 | (22%) | 0.95 |
| Hepatic / Biliary | Local | 894 | 216 | (24%) | 176 | (20%) | 205 | (23%) | 209 | (23%) | 0.19 |
|  | Regional | 718 | 56 | (8%) | 389 | (54%) | 46 | (6%) | 406 | (57%) | 0.56 |
|  | Distant | 249 | <10 |  | 54 | (22%) | <10 |  | 63 | (25%) | 0.62 |
| Esophageal | Local | 345 | 54 | (16%) | 96 | (28%) | 45 | (13%) | 108 | (31%) | 0.52 |
|  | Regional | 393 | <10 |  | 143 | (36%) | <10 |  | 132 | (34%) | 0.09 |
|  | Distant | 228 | <10 |  | 20 | (9%) | <10 |  | 29 | (13%) | 0.37 |
| Anal | Local | 277 | 131 | (47%) | 13 | (5%) | 123 | (44%) | 11 | (4%) | 0.83 |
|  | Regional | 138 | 33 | (24%) | 20 | (14%) | 35 | (25%) | 19 | (14%) | 0.78 |
|  | Distant | 46 | <10 |  | <10 |  | <10 |  | <10 |  | 0.25 |
